# Explaining breastfeeding experiences and assessing factors affecting breastfeeding self-efficacy in mothers of premature infants: a mixed method study protocol

**DOI:** 10.1186/s12978-020-0895-2

**Published:** 2020-03-18

**Authors:** Gholamreza Asadi, Armin Aslani, Anvar-Sadat Nayebinia, Azita Fathnezhad-Kazemi

**Affiliations:** 1 Department of Pediatric, Tabriz Branch, Islamic Azad University, Tabriz, Iran; 2 Student Research Committee, Islamic Azad University, Tabriz branch, Tabriz, Iran; 3 Department of Midwifery, College of Nursing & Midwifery, Clinical Cares and Health Promotion Research Center, Karaj Branch, Islamic Azad University, Karaj, Iran; 4 Reproductive Health, Department of Midwifery, Tabriz Branch, Islamic Azad University, Tabriz, Iran

**Keywords:** Experiences, Breastfeeding, Preterm infants, Social determinants of health

## Abstract

**Background:**

Breastfeeding has a great effect on health promotion and disease prevention in premature infants. However, various factors affect the success of breastfeeding process in mothers. The present study aims to: a) explain breastfeeding experiences; b) assess the factors affecting breastfeeding self-efficacy; and c) present a guideline for promoting breastfeeding in mothers of premature infants.

**Methods:**

This mixed-methods study with a sequential explanatory design consisted of three phases. The first phase is qualitative study to explore the breastfeeding experiences in mothers of premature infants. In this phase, the subjects will be selected through purposive sampling; moreover, in-depth individual interviewing will be used for data collection. Finally, the conventional content analysis approach will be employed for data analysis. The second phase is quantitative and will be used a cross-sectional approach to assess the association of the social determinants of health with breastfeeding self-efficacy in mothers of premature infants. In this phase, the multistage cluster sampling method will be used to select 360 subjects who will be visited healthcare centers in Tabriz, Iran. The third phase focused on developing strategies to increase the ability of mothers to breastfeed their premature infants, using the qualitative and quantitative results of previous phases, a review of the related literature, and the nominal group technique will be performed among experts.

**Discussion:**

The present research is the first study that investigated the experiences of breastfeeding and factors influencing breastfeeding self-efficacy in mothers of premature infants. For the purposes of the study, the mixed methods approach will be used which aimed to develop strategies for the improvement of healthcare services in this regard. It is worth noting that there is no strategic guideline in Iran’s healthcare system for the improvement of breastfeeding, especially regarding mothers of premature infants. Therefore, it is hoped that the strategy proposed in the current study can lead to improvements in this regard.

**Ethical code:**

IR.TBZMED.REC.1398.100.

## Plain English summary

Breastfeeding confers crucial health benefits to both mothers and their babies. Unfortunately, the prevalence and duration of breast milk supply among premature infants tend to be lower than that of full-term infants. The current study provides precise information about the breastfeeding experiences of mothers of premature infants, and the factors influencing them. This study will be used the mixed methods approach with the sequential explanatory design and is comprised of three phases. The first phase is qualitative and explored the views of mothers. The second phase is quantitative and will be used a cross-sectional approach to assess the factors affecting the breastfeeding self-efficacy in mothers of premature infants in Tabriz. The findings of the qualitative and quantitative phases, the literature review, and nominal group technique will be used to establish some strategies to improve and promote breastfeeding practices. The strategy which is proposed by this study may be helpful to increase the ability of breastfeeding and promote health in premature infants.

## Introduction

Breast milk protects one’s health in childhood [1, 2] and also provides health and wellness for the rest of one’s life [3]. The importance of breastfeeding in various respects has been well recognized [4] It has protective effects on maternal and neonatal health, prevents multiple diseases and complications, and as a result reduces healthcare costs [2, 5], Moreover, it helps establish an attachment between a mother and her infant and is a good strategy for the reduction of infant mortality [6]. Many national and international organizations emphasize the importance of breastfeeding [7, 8]. For instance, according to the American Academy of Pediatrics, breastfeeding during the first 6 months is crucial for all the infants, especially premature ones [9] since they have a higher risk of death, complications, and behavioral problems, compared to the term infants [9–11]. These infants require special care and attention, and since they are prone to acute and chronic diseases, breastfeeding is more crucial for them [10]. The data suggest that breastfeeding is more beneficial for these infants and plays an important role in the development of their immune and cognitive systems [8, 12]. However, research has shown that preterm infants are more at risk of not receiving enough milk and that the rate of breastfeeding success in mothers of preterm infants is lower than that in mothers of term infants [5, 10]. This can be due to the fact that these mothers have a vulnerable and fragile situation which can affect their breastfeeding [5]. Moreover, malnutrition due to the lack of knowledge or attitude in mothers of these infants can exacerbate their problems, such as cardiopulmonary instability, premature fatigue during feeding, excessive irritability, prolonged sleep duration, have been reported which are more frequent in them than term neonates [12]. Ultimately, these infants are at higher risk of weight loss and developmental disorders during the early infancy period [13]. Empowerment plays a key role in the promotion of breastfeeding and the identification of the factors that can help improve breastfeeding practices [7]. Previous research suggests that multiple factors can be effective in initiating lactation and continuing the successful breastfeeding process [14–16]. In this regard, the researchers have examined the impact of socioeconomic status, physical-psychological status, social support, and self-efficacy. Given the results of these studies, it seems that any of these factors as social determinants of health can have a positive or negative effect on breastfeeding [8, 16, 17]. These factors are especially highlighted in mothers of preterm infants since they face additional obstacles leading to the delayed onset of breastfeeding [1]. Furthermore, studies show that mothers of preterm infants have lower socioeconomic status, compared to mothers of term infants which, in turn, can have negative effects on breastfeeding [16, 18]. In addition, breastfeeding self-efficacy of women is influenced by their perception of social support as well as their physical and mental status [8]. Therefore, it can be said that a number of different factors contribute to breastfeeding success [10, 19]. One of the strategies for achieving breastfeeding success is focusing on the affective factors; however, the experience of breastfeeding after preterm delivery has not been studied well [1, 10]. Addressing this knowledge gap is very important in the context of caring and support planning. Therefore, research in this field is of particular importance and can be helpful in understanding breastfeeding behavior. Such studies should examine the attitudes and beliefs, affective physical-psychological factors and social norms, and barriers regarding breastfeeding. The present research was designed to explore the factors affecting breastfeeding in mothers of preterm infants and their experiences. Moreover, this study aimed to investigate the impact of social determinants of health on breastfeeding self-efficacy.

### Objectives

The objectives of each phase are as follows:

### Objectives of the first phase: qualitative study

1. To explore breastfeeding experiences among mothers of premature infants
2. To explore views about barriers and facilitators of breastfeeding in mothers of premature infants

### Objectives of the second phase: quantitative study

1. To determine Socio-economic situation among mothers of premature infants.
2. To determine perceived social support among mothers of premature infants.
3. To determine Psychological factors among mothers of premature infants.
4. To determine breastfeeding self-efficacy in mothers of premature infants.
5. To determine the association between Socio-economic situation with breastfeeding self-efficacy.
6. To determine the association between perceived social support with breastfeeding self-efficacy.
7. To determine the association between Psychological factors with breastfeeding self-efficacy.

### Objectives of the third phase

1. To develop Strategies to increase the ability of breastfeeding among mothers of Premature infants

## Methods/design

### Study design

**Fig. 1:**
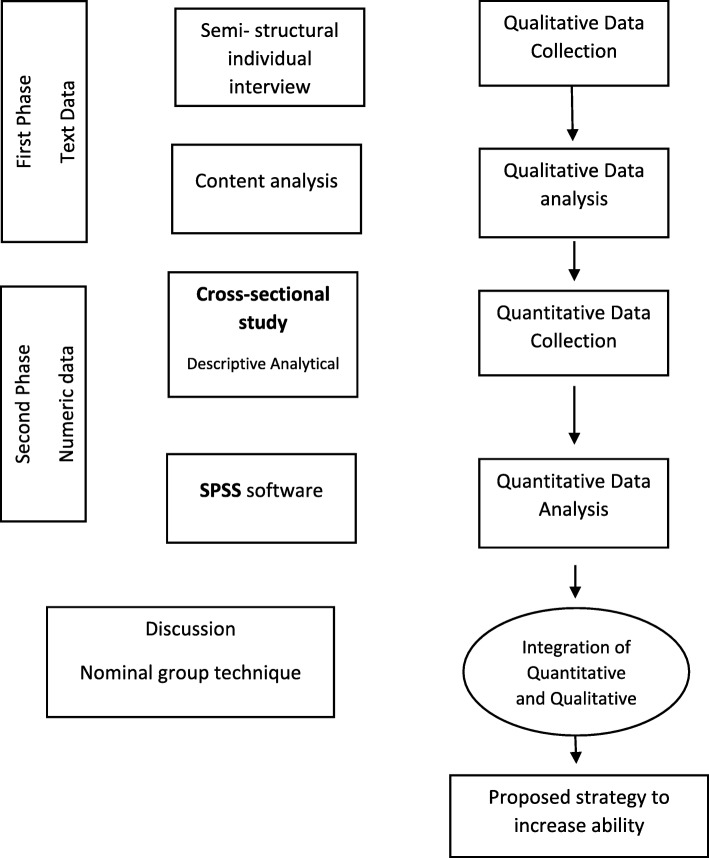
Study diagram

A mixed methods sequential explanatory design will be used to conduct this study by collecting, analyzing, and integrating the qualitative and quantitative data. The mixed-methods paradigm is based on the principles and logic of pragmatism. According to this paradigm, the mixed use of qualitative and quantitative approaches results in a better understanding of the problem [20, 21]. This study will have three phases, and the qualitative and quantitative data will be collected in the first and second phases, respectively. Phase one is an exploratory qualitative study to explore breastfeeding experiences of mothers of premature infants in more detail. Phase two is cross-sectional study to assess the factors affecting the breastfeeding self-efficacy in mothers of premature infants. Phase three is about developing an evidence based and culturally sensitive guideline based on literature review, the results of phase one and two and experts’ opinion using the nominal group technique (Fig.1).

### First phase: qualitative study

First Phase is an exploratory qualitative study with a conventional content analysis approach to explore experiences breastfeeding and views about barriers and facilitators of breastfeeding in mothers of premature infants.

### Sampling method

The research participants will be selected through purposive sampling among mothers of premature infants. Inclusion criteria including: having Iranian nationality and Resident in Tabriz, Ability to understand and convey concepts to the researcher, Mothers who give birth between 28 and 37 gestational weeks, having a history of at least 1–2 months of breastfeeding, having the tendency and ability to express and transfer concepts, with considering the maximum variability in terms of factors such as education, age, socioeconomic status. The withdrawal and non-attendance of the patient were considered as the exclusion criteria.

### Data collection

Qualitative data will be collected using in-depth and semi-structured interviews, containing open questions. Before conducting the interviews, the research team reviews the questions, and the ways to obtain valid data and focus on research questions. The interviewer will pilot the interview on a subset of participants, and used this information to further refine the guide with respect to culturally sensitive and appropriate questions. The interview will begin with a key question, “What were your experiences and feelings about feeding your premature infant?”. Then, the interview will continue by presenting other questions, such as “How do you cope with the difficulties of breast-feeding your baby?”, “What do you think about breastfeeding support? Have you received support?”, “What factors, in your opinion, can be helpful in breastfeeding?” and “What factors do you think may hinder your lactation?”. The interview will continue with more in-depth items, such as “what do you mean? Why? Can you explain further? Can you give an example?” to explore the depth of their experience. During the interview, as far as possible, the Note field will be used and non-verbal data such as tone of voice and behaviors were recorded, too. The sampling will continue until data are saturated. All interviews will be carried out in care centers in a quiet room and without someone other than the interviewees.

### Data analysis

Data analysis process will be performed simultaneously with data collection using MAXQDA software version 10. The qualitative data will be analyzed using qualitative content analysis based on the Graneheim and Lundman method [22]. In this approach, the data will be analyzed through frequent text reading to obtain a full understanding of it. Then, the texts will be read word by word to extract the codes. First, the objective words that contain the key concepts will be specified. The researcher continued digging the text by taking notes from the initial analysis until the major codes will be extracted. In this process, the code labels reflecting more than one key thought will be directly extracted and specified. Then, the codes will be categorized based on their difference and/or relationships. The codes will be categorized into themes and main categories. Subcategories will be extracted based on differences and similarities.

### Validation

To validate the results, at first it will be tried to establish a friendly relationship with the participants. In order to increase the accuracy of the data and for verification of the accuracy of the data, after the registration, the interviews will be given to the participants to review and confirm their stated content and, if there will be any other content, it will be added to the data. Interviews will frequently read by the corresponding author of the paper; then, the text of the interviews with the extracted codes and categories will be shared with the colleagues and their comments will be used. External monitoring will be also used to increase the reliability. By providing the initial code derived from the analysis and examples of the extraction, as external observers, the concepts will be given to other researchers who will be not related to the study in order to determine whether they also will have a similar perception of the data or no.

### Second phase: quantitative study

First, a cross-sectional descriptive analytical study will be conducted to evaluate association of the social determinants of health with breastfeeding self-efficacy in mothers of premature infants.

### Sample size and sampling method

Based on a study conducted by Nego et al. [8], and a confidence coefficient of 95%, statistical power of 90%, standard error of the mean (SEM) of 0.05, mean values of 141.1, 69.3, and 8.3, and the highest standard deviations of 15.9, 9, and 4.6 regarding the breastfeeding self-efficacy scale, perceived social support, and psychological factors and depression the necessary sample sizes were calculated at 20, 26, and 240 subjects, respectively. Since the sample size based on psychological factors and depression was larger, it was selected as the sample size of the study. Regarding the cluster sampling method and a design effect of 1.5, the final sample size was estimated at 360 participants.

Sampling will be conducted in the healthcare centers in Tabriz. There are 82 public healthcare centers (*n* = 40) and posts (*n* = 42) in different regions of the city that provide primary care services, including postpartum care and infant care, free of charge. All the mothers and infants in Tabriz have health records in these healthcare centers and posts. A two-stage cluster sampling will be carried out so that 13 healthcare centers and 14 healthcare posts were selected using a randomizer software (www.random.org). A list of all breastfeeding mothers of premature infants at each center will be extracted from the health records and the samples will be randomly selected from the ordered numbers. Afterward, Using the phone number registered in each record, the researcher will call the potential participants and invite them to participate in the study. The eligible participants will be provided with full explanations about the study objectives and procedures. Also, informed consent will be obtained before collecting information, and be emphasized the importance of honest answers to the questionnaire, and asked them to complete the anonymous questionnaires in a private room. A number of participants are likely to be illiterate, so the questionnaires will be completed by the researcher in order for the data collection method to be the same for all individuals.

### Inclusion criteria

The inclusion criteria consisted of 1) Iranian nationality, 2) residency in Tabriz, 3) literacy, 4) infant age range within 28–37 weeks, 5) passage of 4–6 months after the preterm birth of the infant, 6) lack of disease and malformations of the infants, 7) no history of stressors in the last 6 months (e.g., divorce, death of a family member, and diagnosis of a family member with an incurable or life-threatening disease), 8) breastfeeding (exclusively or with auxiliary milk).

### Exclusion criteria

The exclusion criteria were failure to complete the questionnaire completely and unwillingness to continue the study.

### Scales and data collection

Quantitative data will be collected using 5 questioners, including:

1. **Sociodemographic characteristics questionnaire:** consisted of questions about the mother’s and father’s ages, the mother and father’s education, number of pregnancies, number of deliveries, pregnancy interval, gestational age at delivery, and the infant’s age and sex.
2. **Socioeconomic Status Questionnaire:** This questionnaire had 19 questions about occupational status, level of education of the couple, residence, status of home ownership, the amount of rent or mortgage, house area, family economic status (evaluating eight items), family size, number of family members, ethnicity, insurance, receiving food aid, whether being supported by social organizations, monthly income, and total family expenditure. The economic status will be assessed as poor, average, and good by having less than 3 items, 4 to 6 items, and more than 7 items, respectively. The validity of the researcher-made socioeconomic questionnaire have been evaluated in Dolatian et al. study through content validity, and its reliability have been assessed using internal consistency, Cronbach’s alpha (0.876), and retest (0.916) [23].
3. **Multiple Scale of Perceived Social Support Questionnaire (MSPS):** This is a social support questionnaire designed by Zimet et al. that encompasses 12 items scored based on a Likert scale. The questionnaire evaluates three domains of perceived support from the family (four items), perceived support from family members and acquaintances (four items), as well as perceived support from friends (four items). The items are scored based on a seven-point Likert scale from “completely disagree” (score: 1) to “completely agree” (degree: 7) in which the minimum and maximum scores are 12 and 84, respectively [19]. Total scores of answering the questions fall in three categories: Low (scores 12–48), Moderate (scores 49–68) and high (scores 69–84) social supports [24]. This instrument also has been validated in Iran by Bagherian-Sararoudi R et al., as Cronbach’s coefficient has been found to be 0.92 for the scale and 0.89, 0.92 and 0.87, for friends, significant others and family subscales, respectively [25].
4. **The Depression, Anxiety and Stress Scale (DASS-21):** This is a 21-item questionnaire first presented by Love bound in 1995 that uses 7 questions to measure each of the symptoms of stress, anxiety, and depression. This questionnaire was designed as a Likert questionnaire. The lowest score for each question was a score of zero and the highest the score of 3. In each section related to anxiety, depression and stress, a score of 1–7 indicated a mild level, a score of 8–14 indicated a moderate level, and a score of 15–21 indicated a severe level of anxiety, depression and stress. This questionnaire has been used in various surveys and its validity and reliability have been confirmed [23].
5. **The breastfeeding self-efficacy questionnaire**: It was designed by Dennis (2003). This questionnaire is a 33-item scale and is scored by a 5-point Likert scale from always confident (5) to not at all confident (1). Scores range from 33 to 165, with 33–76 considered low self-efficacy, 77–120 average self-efficacy, and 121–165 regarded as high self-efficacy. The reliability of this questionnaire was reported 82% in a study by Varaei et al. [26].

### Data analysis

The quantitative data will be analyzed with SPSS-22. Sociodemographic, MMAS-8, DASS-21, self-efficacy breastfeeding questionnaires score will be described by frequency (percent), as well as mean (standard deviation) if the data are normally distributed. The relation between Sociodemographic, MMAS-8, DASS-21 with self-efficacy breastfeeding will be determined using the independent test, ANOVA and Pearson correlation tests in the bivariate analysis, and logistic linear regression adjusting the confounding variables in the multivariate analysis. Then, the univariate logistic regression will be used to control confounding variables.

### Third phase: integration of quantitative and qualitative data

To develop improvement strategies for increase ability of breastfeeding in mothers of premature infants, a comprehensive literature review will be carried out with a supportive approach to improve such practices. Following this, the results from qualitative and quantitative studies will be delivered to 10–12 experts. Then, their feedback and comments will be taken into account, using the nominal group technique.

## Discussion

The prevalence and duration of breastfeeding the premature infants tend to be lower than that of full-term infants [10], while feeding human milk to preterm infants may impact their long-term health and development [3, 5]. However, the mothers of premature infants face various problems in the process of breastfeeding due to different causes that can lead to its interruption and cause problems, which have repercussions on the health and well-being of the infants [10, 12, 16]. Very few studies have focused on these problems and psychosocial needs and opinions of mothers. Furthermore, listening to the mothers, and considering the affective factors on breastfeeding is a core need for quality family-centered care and can also help establish appropriate strategies. Therefore, the importance of breastfeeding and its affective factors should be recognized. It is also worth noting that there is no strategic guideline in Iran’s healthcare system for the improvement of breastfeeding in mothers of premature infants. The current study provides precise information about breastfeeding experiences, its related factors, barriers, and facilitators. The present research is the first one to investigate factors influencing breastfeeding which was performed using the mixed methods approach, and aimed to develop health practices improvement strategies. Data collection through qualitative and quantitative methods contributed to a better comprehension of the factors influencing breastfeeding. The mixed-methods approach focused on epistemological pluralism. As a result, this study made use of a combination of opinions, approaches, and different, even contradictory, methods to achieve a better understanding of the related concepts. The strategy proposed by this study may help healthcare professionals and policymakers to become aware of the factors influencing breastfeeding in mothers of premature infants, improve breastfeeding ability, and help to promote health in premature infants. Therefore, it is hoped that the strategy proposed in the current study leads to the improvement of breastfeeding practices in mothers of premature infants.

## Data Availability

Not applicable.

## Abbreviations

DASS-21: The Depression, Anxiety and Stress Scale
MSPS: Multiple Scale of Perceived Social Support Questionnaire
SES: Socioeconomic Status
